# Burnout syndrome in university professors and academic staff members: psychometric properties of the Copenhagen Burnout Inventory–Brazilian version

**DOI:** 10.1186/s41155-020-00151-y

**Published:** 2020-06-28

**Authors:** Fernanda Ludmilla Rossi Rocha, Lilian Carla de Jesus, Maria Helena Palucci Marziale, Silvia Helena Henriques, João Marôco, Juliana Alvares Duarte Bonini Campos

**Affiliations:** 1 Escola de Enfermagem de Ribeirão Preto, Universidade de São Paulo – EERP/USP, Avenida Bandeirantes, 3900, Ribeirão Preto, São Paulo, 14040-902 Brazil; 2 Instituto Universitário de Ciências Psicológicas, Sociais e da Vida – ISPA, Rua Jardim do Tabaco 34, Alfama, 1100-304 Lisboa, Portugal; 3 Faculdade de Ciências Farmacêuticas de Araraquara, Universidade Estadual Paulista “Julio de Mesquita Filho” – FCFAr/UNESP, Rodovia Araraquara Jaú, Km 01 - s/n - Campos Ville – Araraquara, São Paulo, 14800-903 Brazil

**Keywords:** Occupational health, Professional burnout, Copenhagen burnout inventory, Validation, Psychometrics

## Abstract

The aims of this study were to evaluate the psychometric properties of the Copenhagen Burnout Inventory–Brazilian version (CBI-Br) in a sample of university professors and academic staff members of Brazilian public universities, to estimate the level of burnout syndrome (BS) among these workers, and to assess the associations of BS with demographic and occupational determinants of the syndrome. A total of 676 workers participated in the study. Confirmatory factor analysis results supported a three-factor model with 18 items and an acceptable overall fit. Adequate convergent and discriminant validity of the CBI-Br’s factors were observed, as well as adequate reliability of the instrument for the sample. In conclusion, the results of this study provide evidence of the validity and reliability of the CBI-Br for the measurement of BS in Brazilian university professors and academic staff members. In addition, the CBI-Br may be an important tool for the diagnosis of psychosocial risks related to BS in the academic environment.

## Introduction

The concept of burnout was introduced in the literature in the 1970s by Freudenberger (1974) and Maslach (1976); it was based on a social-psychological perspective and was described as “a syndrome of emotional exhaustion and cynicism that occurs frequently among individuals who do ‘people-work’ of some kind. A key aspect of the burnout syndrome is increased feelings of emotional exhaustion” (Maslach & Jackson, 1981, p.99). Currently, burnout is considered a prolonged response to chronic emotional and interpersonal stressors on the job (Maslach, Schaufeli, & Leiter, 2001).

BS is considered a state of physical and emotional exhaustion caused by long-term involvement in emotionally demanding situations (Schaufeli & Greenglass, 2001). In addition, BS is defined as a combination of physical and emotional exhaustion caused by different work demands (Schaufeli & Bakker, 2004), which represent organizational aspects that require continuous physical, cognitive, or emotional worker’s effort (Karasek & Theorell, 1990; Demerouti, Bakker, Nachreiner, & Schaufeli, 2001). Therefore, BS has been directly related to occupational stressors, defined as work-related conditions that generate tension arising from the imbalance between work demands and workers’ perceptions of their ability to deal with these demands appropriately (Leka, Griffths, & Cox, 2003).

In the 1980s, Maslach and Jackson proposed an instrument to the assessment of burnout: the Maslach Burnout Inventory (MBI; Maslach & Jackson, 1981). The MBI has been recognized as the most widely used instrument to evaluating burnout syndrome (BS). However, during the last two decades, researchers have discussed the theoretical concept and methodological aspects of the MBI (Demerouti et al., 2001; Kristensen, Borritz, Villadsen, & Christensen, 2005), considering an unclear relationship between the burnout concept and the MBI and a psychometric shortcoming of MBI related to the items framework (Demerouti, Bakker, Vardakou, & Kantas, 2003; Milfont, Denny, Ameratunga, Robinson, & Merry, 2008).

Considering exhaustion as the core of the burnout concept, researchers from Denmark developed the Copenhagen Burnout Inventory (CBI) (Kristensen et al., 2005). The CBI is composed of 19 items distributed in three subscales measuring personal burnout, work-related burnout, and client-related burnout, which represent the degree of physical and psychological exhaustion experienced by the individual as related to his/her own life and work (Kristensen et al., 2005).

The CBI has been translated and adapted in different countries, such as China (Yeh, Cheng, Chen, Hu, & Kristensen, 2007), New Zealand (Milfont et al., 2008), Brazil (Campos, Zucoloto, Bonafé, Jordani, & Marôco, 2011), Portugal (Fontes, 2011; Campos, Carlotto, & Marôco, 2013), Spain (Campos et al., 2013), Spain (Molinero, Basart, & Moncada, 2013),Italy (Avanzi, Balducci, & Fraccaroli, 2013), Serbia (Berat, Jélic, & Popov, 2016), Thailand (Phuekphan, Aungsuroch, Yunibhand, & Chan, 2016), Iran (Mahmoudi et al., 2017), and Malaysia (Andrew Chin et al., 2018).

Like the MBI, the CBI has been used to analyze the BS in human services workers (Kristensen et al., 2005; Molinero, Basart, & Moncada, 2013; Berat et al., 2016), health professionals (Chou, Li, & Hu, 2014; Chin et al., 2018), university students (Marôco & Campos, 2012; Campos et al., 2013), and university professors and academic staff (Kinman & Wray, 2014; Milfont et al., 2008; Avanzi et al., 2013; Fiorilli et al., 2015; Sestili et al., 2018).

Studies developed by the Health and Safety Executive of the UK over the last decades indicate that work-related stress is increasing in higher education institutions, with serious implications for workers’ health and wellbeing (Kinman, 2014; Kinman & Wray, 2014).

Teacher burnout has been studied since the 1990s in terms of work conditions (Kyriacou, 2001; Schaufeli, Leiter, & Maslach, 2009) and the consequences of burnout for educators’ health and efficiency at work and for student outcomes (Zhong et al., 2009; Skaalvik & Skaalvik, 2010; Brunsting, Sreckovic, & Lane, 2014). A review of burnout predictors in university professors identified some stressful factors that can trigger emotional exhaustion and low job satisfaction: work pressure, low pay, low social recognition, conflicts at work, problematic relationships with parents, large classes, and learning difficulties and aggressive behaviors in students (Fiorilli et al., 2015). University professors must develop multiple activities for teaching and research projects as well as attending to scientific production requirements and performing administrative tasks (organizing departments and faculties, planning academic activities, managing courses) (Sestili et al., 2018). In addition, the combined effect of the response to job demands with the progressive degradation of work conditions at universities worldwide results in physical and emotional exhaustion among professors (Collado, Soria, Canafoglia, & Collado, 2016).

Regarding academic staff burnout, research interest has been growing significantly since the 2000s (Winefield et al., 2003; Kinman, 2008; Kinman & Court, 2010; O’Connor & O’Hagan, 2016). Work intensity and long working hours were identified as specific negative determinants for work-life balance among academic employees (Hogan, Hogan, Hodgins, Kinman, & Bunting, 2014), as well as high levels of stress due to time pressure, workload, poor remuneration, feelings of job insecurity, and reduced clarity of role expectations (Poalses & Bezuidenhout, 2018). These results corroborate studies which correlate occupational stress with BS among academic staff members (Adekola, 2012; Mark & Smith, 2012; Khan & Yousaf, 2016; Nazari et al., 2016*)*.

Despite this context, to study the relationships of burnout with established job stressors or psychosocial factors among university professors and academic staff members, including age, gender, work function, hours worked per week, and duration of employment, become relevant. Therefore, the aims of this study were (i) to evaluate the psychometric properties of Copenhagen Burnout Inventory (CBI) in a sample of university professors and academic staff members of Brazilian public universities, (ii) to estimate the level of BS among these workers, and (iii) to assess the magnitude of associations of the level of BS with known determinants from scientific evidence.

## Method

### Study design and sample

This is a methodological study developed to evaluate the psychometric properties of the CBI-Br. The data collection was performed using an observational cross-sectional design and a non-probabilistic (convenience) sampling method. Professors and academic staff members at four Brazilian public universities (University of São Paulo (USP), São Paulo State University (UNESP), University of Campinas (UNICAMP), and Federal University of São Carlos (UFSCar)) were invited by email to participate. A total of 8400 emails were sent, but only 905 workers voluntarily agreed to participate (adherence rate = 10.8%). A total of 676 questionnaires were completed and were included in the sample (response rate = 74.7%). Reminders were sent out to respondents for three times, once a month.

The estimated minimum sample size was based on the recommendations of Hair, Black, Babin, Anderson, and Tatham (2005)), who consider 5–10 subjects necessary per parameter (*k*) to be estimated by the model. Because the instrument has 41 parameters (19 items, 19 errors, and 3 correlations between factors), it was estimated that 205 to 410 participants would be required. Considering the possibility of a dropout rate of approximately 20%, the minimum required sample size was increased to 257 to 513 subjects. In addition, to assess the invariance of the factorial model, a second sample of the same size was necessary. Participants who did not respond to all items of the instrument were excluded.

The mean age of the sample was 48.05 years (SD = 10.66, range = 18–79, quartile 1 = 40.5, quartile 3 = 57.5, median = 49), 56.2% (*n* = 380) were women, and 54.7% (*n* = 370) were professors. Regarding the duration of employment, 380 (56.2%) participants had worked for up to 15 years at the universities, and 94.6% (*n* = 639) of the sample worked full time or 40 h per week.

### Instruments

The instruments were made available for online completion through an electronic survey platform (*SurveyMonkey*) for 6 months (from May to October 2018). For the sample characterization, a demographic questionnaire with information related to the workers’ gender, age, position at work, duration of employment at the universities, and hours worked per week was used.

To assess BS, the Copenhagen Burnout Inventory (CBI) (Kristensen et al., 2005) was used. The 19 items of the CBI are rated on a 5-point scale from 1 (never) to 5 (always) or from 0 to 100 points, with high scores indicating high levels of burnout. However, the original authors do not offer cutoff points for scoring the instrument. The items are distributed in three subscales measuring physical and psychological fatigue associated with personal burnout (PB), 6 items; work-related burnout (WB), 7 items; and client-related burnout (CB), 6 items. Item 10 of the WB subscale has an inverted response scale in relation to the other items of the CBI. The authors provided formal authorization for the use of the scale.

According to Kristensen et al. (2005), the PB subscale refers to the degree of physical and psychological fatigue and exhaustion experienced by the respondent. The WB subscale represents the degree of physical and psychological fatigue and exhaustion perceived by the respondent as related to his/her work. The CB subscale is defined as the degree of physical and psychological fatigue and exhaustion perceived by the respondent as related to his/her work with clients. The authors state that “clients” is a broad concept that can be adapted to specific groups of respondents when the CBI is used in practice (e.g., the respondents’ students or workers) (Kristensen et al., 2005).

Since its development, the CBI has demonstrated robust psychometric properties (Kristensen et al., 2005; Yeh et al., 2007; Milfont et al., 2008; Campos et al., 2011; Avanzi et al., 2013; Molinero et al., 2013; Phuekphan et al., 2016; Andrew Chin et al., 2018) for analyzing BS even when applied to different populations. The cultural adaptation of the CBI into Brazilian Portuguese was performed by Campos et al. (2011). They adapted the original instrument for a sample of Brazilian university students to create the CBI-student version (CBI-SS).

### Ethical consideration

The present study was approved by Research Ethics Committee (CAAE 5477715.1.0000.5393). It was followed ethical regulations established by Resolution 466/2012 of the Brazilian National Health Council.

### Evidence based on test content

In this study, the CBI-SS (Campos et al., 2011) was adapted to evaluate BS in a sample of university professors and academic staff members. The original three-factor CBI was maintained, but the subscales now comprised of personal burnout (PB), work-related burnout (WB), and *colleague*-related burnout (CB). The term “clients” was replaced with “colleagues” considering the population (university professors and academic staff members) and scientific evidence related to occupational stress and burnout predictors (Kinman, 2008; Khan, Din, & Anwar, 2019). These studies demonstrated that poor working relationships have frequently been highlighted as stressful aspects of academic work.

This process represented the content validity of the instrument and was carried out by a Committee of Experts composed of three university professors (researchers in occupational health, management, and education) and three members of the academic staff of a Brazilian public university. The committee members analyzed the original versions of the CBI and the CBI-SS and evaluated the idiomatic, semantic, cultural, and conceptual equivalences of the instrument proposed in this study, named the CBI-Brazilian version (CBI-Br), suggesting minor revisions.

To analyze the experts’ evaluation, we used a content validity index (CVI), a 4-point scale based on ratings of item relevance (1 = not relevant, 2 = somewhat relevant, 3 = quite relevant, 4 = highly relevant). For each item, an item-level content validity index (item CVI) was computed by dividing the number of experts who gave the item a rating of 3 or 4 by the total number of experts (proportion of relevance agreement). Then, the average of the item indices and the scale-level content validity index average were computed. An instrument is determined to have excellent content validity if the item CVI ≥ 0.78 and the scale CVI ≥ 0.90 (Waltz, Strickland, & Lenz, 2005).

### Data analysis

The psychometric properties of the CBI-Br were analyzed by estimating the psychometric sensitivity of the items; the factorial, convergent, and discriminant validity; the factorial invariance; and the reliability of the model (Fornell & Larcker, 1981; Marôco, 2014).

The psychometric sensitivity was determined using summary (mean, median, and standard deviation) and form (skewness and kurtosis) measures of items responses. Sensitivity was considered adequate when the distribution of the response frequencies approximated a normal curve, and the absolute values of skewness and kurtosis were less than 3 and 7, respectively (Nunnally, 1978; Marôco, 2014). The multivariate normality of the data was assessed by Mardia’s test (adequate value < 3.0).

Factorial validity was determined using confirmatory factor analysis (CFA) with the maximum likelihood (ML) estimation method. To evaluate the goodness of model fit, the *x*^2^/df (ratio of chi-square and degrees of freedom), comparative fit index (CFI), Tucker-Lewis index (TLI), and root mean square error of approximation (RMSEA) were used. Model fit was considered acceptable when *x*^2^/df ≤ 5.0, CFI and TLI ≥ 0.90, and RMSEA ≤ 0.10 (Tanaka & Huba, 1985; Bentler, 1990; Arbuckle, 2008). Factor weights (*λ*) were considered appropriate when they were ≥ 0.50 (Hair et al., 2005). The modification indices were calculated using the Lagrange multipliers (LM) method to inspect the need for model refinement, considering values of LM > 11 (Marôco, 2014). A second-order hierarchical model (SOHM) was also tested, with burnout as the second order factor.

To evaluate the convergent validity of each CBI-Br subscale, the average variance extracted (AVE) was estimated. Evidence of convergent validity was assumed if AVE ≥ 0.50 (Fornell & Larcker, 1981). Discriminant validity was accepted when the AVE for each factor was larger than the squared Pearson correlation between the two factors (AVE_i_ and AVE_j_ ≥ ρ_ij_^2^) (Fornell & Larcker, 1981).

Factorial invariance between independent samples was evaluated to verify the external validity of the obtained factorial solution using multigroup cross-validation analysis and the chi-square difference statistical test (Δ*x*^2^). For this purpose, the sample was randomly divided into two independent samples (test sample: *n* = 338; validation sample: *n* = 338). To evaluate invariance, the factorial weights (*λ*), intercepts (*i*), and residual variance/covariance (Cov) of the two samples were analyzed. When *p*Δ*x*^2^_**λ**_ was > 0.05, weak invariance (metric) was found; if *p*Δ*x*^2^_**λ**_ and *p*Δ*x*^2^_i_ were > 0.05 (metric and scalar invariance) or *p*Δ*x*^2^_**λ**_, *p*Δ*x*^2^_i_, and *p*Δ*x*^2^_cov_ were > 0.05 (metric, scalar, and strict invariance), strong invariance was found.

The reliability of the items was estimated using Cronbach’s *α* and composite reliability (CR). It was considered adequate when *α* and CR ≥ 0.70 (Fornell & Larcker, 1981).

### Overall score of the CBI-Br

The overall score of the instrument was calculated using the matrix of the factor score weights obtained through the CFA. The scores were calculated for both first-order and second-order factors. To maintain the exact metric of the original items of the instrument, the proportion of the contribution of each item to the overall score was used to correct the original factor score weights. The corrected weights were multiplied by each participant’s item response, and the estimated scores of each item were added to obtain the overall score for each factor (overall weighted scores).

### Known-groups analyses

The correlation between age, hours worked per week, duration of employment at the universities, and burnout was estimated according to gender using Pearson’s correlation coefficient (*r*). The correlation between gender, work function (professors and academic staff), and burnout was performed using analysis of variance (ANOVA). The data homoscedasticity assumption was verified (Levene’s test). If the homoscedasticity assumption was rejected, Welch’s correction was used. The significance level was 5%.

### Mean scores of BS in the sample

The mean scores of BS in the sample were calculated using the recommendations of Kristensen et al. (2005): the scale labels were recoded to the format of 1 = 0 (never), 2 = 25, 3 = 50, 4 = 75, and 5 = 100 (always) so that higher scores indicate more burnout.

Statistical analyses were performed using the IBM SPSS Statistics 22 (IBM Corp., Armonk, N.Y., USA) and AMOS 22.0 (IBM Corp., Armonk, N.Y., USA) software.

## Results

Regarding the CBI-Br, the content validity of the instrument was considered excellent because the six experts considered the 19 items relevant (CVI = 100%). However, some adjustments were suggested by using Portuguese synonyms for the words “worn out” (item 5), “leisure time” (item 10), “exhausting” (item 11), and “burnt out” (item 13). The CBI-Br is shown in the Appendix.

**Fig. 1:**
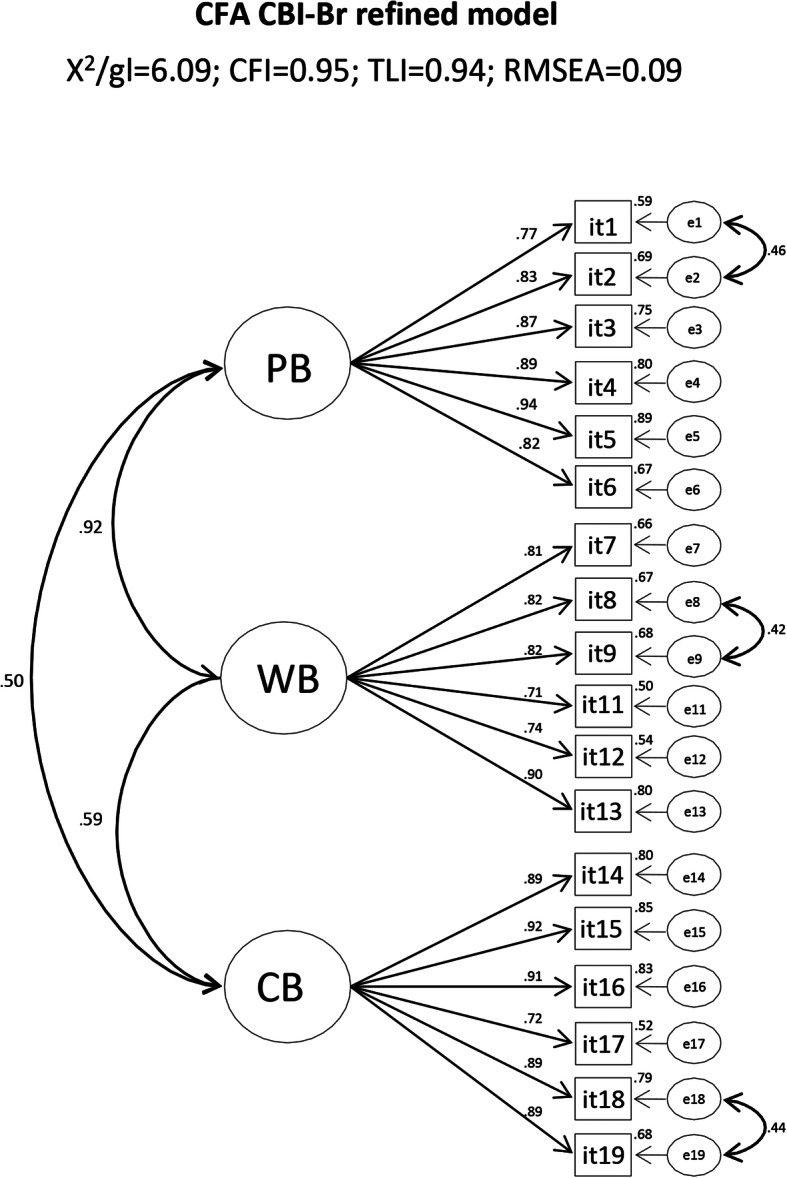
Confirmatory factor analysis of CBI-Br refined model (*x*^2^/df = 6.09; CFI = 0.95; TLI = 0.94; RMSEA = 0.09; IC90% = [0.081–0.093])

The psychometric sensitivity analysis of the CBI-Br items indicated that all the items presented skewness and kurtosis values close with to a normal distribution (Mardia’s test = 2.17). The CFA indicated a poor fit of the CBI-Br to the sample (*x*^2^/df = 7.87; CFI = 0.92; TLI = 0.90; RMSEA = 0.10). The analysis of standardized factorial weights showed that item 10 (the only inverted item) presented *λ* = 0.202; therefore, this item was removed. In addition, based on the modification indices, correlations were inserted between errors (e1–e2 [LM = 128.36], e8–e9 [LM = 91.61], e18–e19 [LM = 87.11]). The CFA of the CBI-Br refined model is presented in Fig. 1.

The refined model resulted in a three-factor model with 18 items, factorial weights (*λ*) > 0.70, moderate to strong factor correlations (*r*_(PBXCB)_ = 0.50, *r*_(WBXCB)_ = 0.59, *r*_(BPXWB)_ = 0.92), and an acceptable overall fit (*x*^2^/df = 6.09; CFI = 0.95; TLI = 0.94; RMSEA = 0.09; IC90% = [0.081–0.093]).

**Fig. 2:**
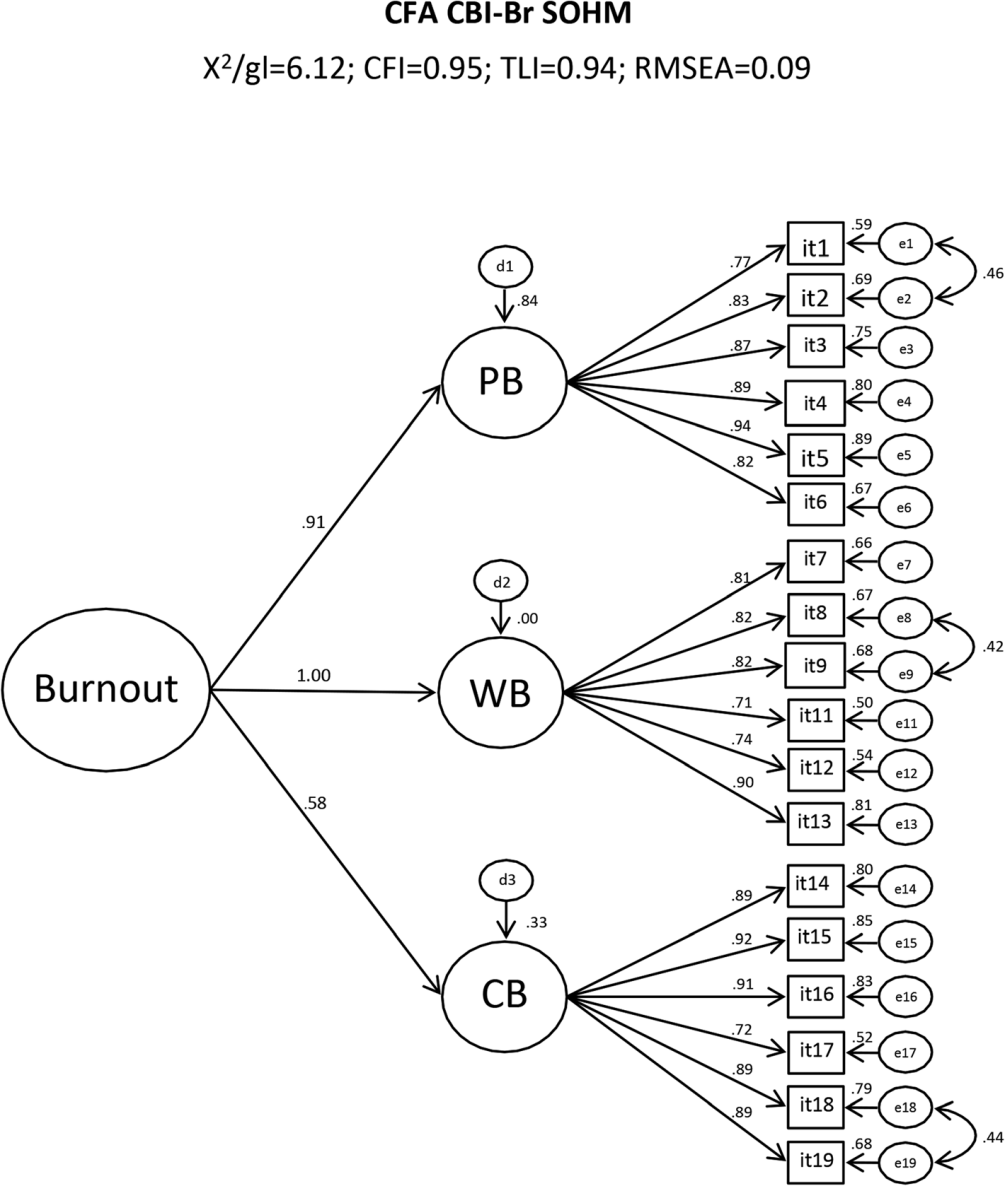
Confirmatory factor analysis of CBI-Br second-order hierarchical model (*x*^2^/df = 6.12; CFI = 0.95; TLI = 0.94; RMSEA = 0.09; IC90% = [0.081–0.093])

Adequate convergent validity for all CBI-Br factors (AVE = 0.64–0.74) was observed. Discriminant validity was verified between AVE_(PB)_ and AVE_(CB)_ (*r*^2^ = 0.25) and AVE_(CB)_ and AVE_(WB)_ (*r*^2^ = 0.35) but not between AVE_(WB)_ and AVE_(PB)_ (*r*^2^ = 0.84). The composite reliability (CR) and the standardized Cronbach’s *α* of the CBI-Br domains were adequate (CR = 0.91–0.94 and *α* = 0.91–0.95), showing adequate reliability of the instrument between the samples. The CFA of the SOHM is presented in Fig. 2.

The SOHM also showed an acceptable fit to the data (*x*^2^/df = 6.12; CFI = 0.95; TLI = 0.94; RMSEA = 0.09; IC90% = [0.081–0.093]) and a strong contribution of the factors PB (*β* = 0.91) and WB (*β* = 1.00) to the general concept of burnout.

Regarding the factorial invariance of the refined CBI-Br model in independent samples (test vs. validation), simultaneous analysis showed the goodness of model fit (*x*^2^/df = 3.59; CFI = 0.95; TLI = 0.94; RMSEA = 0.06) and the metric and scalar invariance of the model (*strong invariance*) (Δ*x*^2^: *λ* = 7.18, *p* = 0.95; *I* = 24.73, *p* = 0.13; Cov = 8.03, *p* = 0.24; residual = 39.93, *p* = 0.01). The CFA, convergent validity, and reliability of the CBI-Br for different samples are presented in Table 1.

**Table 1:** Confirmatory factor analysis (CFA), convergent validity, and reliability of the CBI-Br to different samples

Sample	***λ***	***x***^**2**^/df	TLI	RMSEA	AVE	CR	Α
					PB, WB, CB	PB, WB, CB	PB, WB, CB
Total	0.20–0.94	7.87	0.90	0.10	–	–	–
Total (refined)	0.71–0.94	6.09	0.94	0.09	0.73, 0.64, 0.74	0.94, 0.91, 0.95	0.94, 0.91, 0.95
SOHM	0.70–0.94	6.12	0.94	0.09	0.73, 0.64, 0.74	0.94, 0.91, 0.95	0.94, 0.91, 0.95
Test	0.70–0.95	3.91	0.93	0.09	0.73, 0.64, 0.75	0.94, 0.91, 0.95	0.94, 0.91, 0.95
Validation	0.71–0.94	3.27	0.94	0.08	0.74, 0.64, 0.74	0.94, 0.92, 0.94	0.95, 0.92, 0.94
Validation vs. test	0.70–0.95	3.59	0.94	0.06	–	–	–

*λ* factorial weights, *x*^2^/df chi-square by degrees of freedom, *CFI* comparative fit index, *TLI* Turkey-Lewis index, *RMSEA* root mean square error of approximation, *AVE* average variance extracted, *CR* composite reliability, *α* Cronbach’s alpha coefficient, *SOHM* second-order hierarchical model, *PB* personal burnout, *WB* work-related burnout, *CB* colleagues-related burnout

The ANOVA of the overall weighted scores of the CBI-Br factors (PD, WB, and CB) and burnout (SOHM) between gender and work function at the universities is available in Table 2.

**Table 2:** The ANOVA of the overall weighted scores of CBI-Br (three-factor model) and the burnout (SOHM) between gender and function

Variable	PB	WB	CB^a^	Burnout
**Gender**	(mean ± standard deviation)			
Men (*n* = 296)	2.11 ± 0.90	2.08 ± 0.86	2.05 ± 0.97	2.08 ± 0.86
Women (*n* = 380)	2.68 ± 1.05	2.55 ± 0.97	2.30 ± 0.96	2.55 ± 0.98
Total (*n* = 676)	2.43 ± 1.03	2.35 ± 0.95	2.19 ± 0.97	2.34 ± 0.96
*F* statistic	56.01	43.19	10.91	42.83
*p value*	< 0.001	< 0.001	0.001	< 0.001
**Function**				
Professors (*n* = 370)	2.36 ± 0.96	2.30 ± 0.88	2.24 ± 0.96	2.30 ± 0.89
Academic staff (*n* = 306)	2.51 ± 1.11	2.40 ± 1.03	2.12 ± 0.99	2.40 ± 1.03
Total (*n* = 676)	2.43 ± 1.03	2.35 ± 0.95	2.19 ± 0.97	2.34 ± 0.96
*F* statistic	3.48	1.88	2.62	1.96
*p value*	0.063	0.171	0.106	0.162

*PB* personal burnout, *WB* work-related burnout, *CB* colleagues-related burnout

^a^ANOVA with Welch’s correction

The ANOVA results showed that gender was a social determinant of BS, with statistically significant differences between men and women. Women presented the highest scores, and the work function performed at the universities did not represent a work-related determinant of BS because there was no statistically significant difference between the overall weighted scores of professors and academic staff.

Because of the differences in scores between men and woman, we calculated correlations between age, hours worked per week, duration of employment at the universities, and burnout (SOHM) separately by gender (Table 3).

**Table 3:** Pearson’s correlation matrix between age of participants, hours worked per week, duration of employment, and burnout by gender

Variables	PB	WB	CB	Burnout	Duration	Hours	Age
**Men**							
Duration	− 0.08	− 0.09	− 0.00	− 0.09	1		
Hours	− 0.08	− 0.06	0.01	− 0.06	0.00	1	
Age	− 0.27^**^	− 0.27^**^	− 0.12^*^	− 0.27^**^	0.33^**^	0.14^*^	1
**Women**							
Duration	− 0.08	− 0.08	− 0.08	− 0.07	1		
Hours	− 0.11^*^	− 0.10^*^	− 0.04	− 0.10^*^	− 0.01	1	
Age	− 0.32^**^	− 0.33^**^	− 0.24^**^	− 0.32^**^	0.38^**^	0.21^**^	1

*hours* hours worked per week, *duration* duration of employment at the universities, *PB* personal burnout, *WB* work-related burnout, *CB* colleagues-related burnout

**p ≤ 0.01; *p < 0.05

There were statistically significant negative correlations (*p* < 0.01) between age and PB, WB, CB, and burnout scores, as well as between hours worked per week, PB, WB, and burnout for women (*p* < 0.05).

To describe the levels of BS in this sample, we use the scoring on the 0–100 scale, which results in mean scores of PB = 31.49, WB = 27.06, CB = 25.21, and the overall score of the CBI-Br = 27.92.

## Discussion

This study attested to the validity and reliability of the CBI-Brazilian version (CBI-Br) when applied to a sample of professors and academic staff members at Brazilian public universities and to the relation between gender and work duration and to the level of BS in the sample.

The establishment of the CBI-Br’s content validity was the first step in analyzing the psychometric properties of the instrument. Content validity was considered very satisfactory after the minor changes suggested by experts were made.

Regarding the CBI-Br’s construct validity, the refined model presented three factors and 18 items, a good overall fit and strong invariance in the simultaneous analysis of independent samples. The CFA showed a low factorial weight for item 10, which was excluded. An adequate convergent validity was verified for all domains, and the discriminant validity was not observed between the PB and WB domains.

The low factor weight of item 10 was also observed in other studies (Yeh et al., 2007; Campos et al., 2011; Marôco & Campos, 2012; Campos et al., 2013; Fong, Ho, & Ng, 2014; Fiorilli et al., 2015; Andrew Chin et al., 2018), and it can be attributed to the reverse elaboration of the item. According to Yeh et al. (2007), the CBI items have a pattern of a negative response direction, which creates a stereotype of responses. Because item 10 is the only one with a positive formulation, participants may not notice the difference and may maintain the response pattern. Other authors (Suárez-Álvarez et al., 2018) discussed the effect of wording the items in the same or different directions as a shortcoming of the MBI. Highlight the exclusion of the item did not affect the theoretical assumptions of the instrument.

The absence of discriminant validity between the PB and WB subscales of CBI-Br can be explained by the theoretical approximation between these factors. Although the PB items refer exclusively to the *personal exhaustion* and the WB items reflect only the *work-related exhaustion* experienced by the participant (Kristensen et al., 2005), both PB and WB assess the degree of exhaustion of the individual. Despite the fact that most workers spend most of their lives at work today, as the participants of this study, who work more than 8 h a day, such schedules can hinder them from separating personal and work-related perceptions of exhaustion. These theoretical aspects also justify the strong correlations between these factors, as reported by previous evidences (Yeh et al., 2007; Fong et al., 2013).

The internal consistency of the CBI-Br confirmed the results of other reported studies (Milfont et al., 2008; Marôco & Campos, 2012; Avanzi et al., 2013; Fiorilli et al., 2015; Phuekphan et al., 2016; Mahmoudi et al., 2017; Andrew Chin et al., 2018). In addition, the analysis attested to the strong measure invariance of the refined CBI-Br model between independent samples, indicating invariance of the model and external validity of the factorial structure proposed for the sample.

Regarding the professional variables related to BS, the analysis of the overall weighted scores of CBI-Br factors (PD, WB, and CB) and burnout (SOHM) with gender, job function, age, hours worked per week, and duration of employment allowed us to identify some determinants in the sample, considering the concept of BS.

The gender analysis showed that women presented the highest levels of burnout in the sample. Female gender has been associated with a high burnout risk due to several psychosocial factors: the double duties of home and work, societal gender-related roles and social expectations, the risks of sexual harassment at work and domestic violence, and gender-based discrimination (International Labor Organization [ILO], 2016).

Related to the job function performed at the universities, although professors are among the most frequently investigated professional categories in burnout studies (Carlotto & Câmara, 2017), the association between job function and BS was not significant. Additionally, the number of BS studies among academic staff has increased in recent years (Kinman, 2014), but the role was not considered an occupational determinant of the sample.

There were statistically significant negative correlations between age and PB, WB, and CB, and burnout scores demonstrate that younger workers were the most affected by BS. The younger professors and academic staff members were also the workers with fewer hours worked per week and duration of employment at the universities. Besides, it was observed that the more hours worked per week, the lower the level of PB, WB, and burnout among younger women.

These results corroborate the findings of Marôco et al. (2016), who investigated BS in health professionals at different Portuguese hospitals. The higher level of BS in younger professionals with shorter employment durations may be related to these workers’ lack of positive coping strategies to deal with personal/professional stressors and to individual characteristics. Whether in personal life or in a work environment, positive coping strategies can reduce stress (Janke & Erdmann, 2008).

The analysis of BS in the sample showed that the mean scores of personal, work-related, and colleagues-related burnout were lower than those demonstrated previously by scientific evidence. Kristensen et al. (2005) found average scores of PB = 35.9, WB = 33.0, and CB = 30.9, in different professionals. Milfont et al. (2008) carried out their study in order to evaluate BS in New Zealand secondary school teachers and identified mean scores of 43.0, 41.5, and 40.4 for PB, WB, and CB, respectively. Sestili et al. (2018) identified mean scores of PB = 41.4 and WB = 34.3 (the mean score of CB was not mentioned by the authors). Additionally, they observed that personal and work-related burnout levels were higher in women, younger, and part-time professors, corroborating other results of this study. These evidences also indicate that personal life-related aspects represent decisive predictors for BS, pointing to the need to promote individual coping strategies.

## Limitations

There were some limitations to this study. The cross-sectional design does not allow the establishment of causality effects. The non-probabilistic sampling method and the impossibility of including a larger number of universities hinder the generalization of the results. To minimize these limitations, an extended sample size was used.

## Conclusion

The results of this study provide evidence of the validity and reliability of CBI-Br for the measurement of BS in Brazilian university professors and academic staff members. In addition, the CBI-Br may represent an important tool for the diagnosis of psychosocial risks related to BS in the academic environment. Furthermore, the use of the CBI-Br in the organizational context can support the implementation of preventive measures for burnout and health promotion at work. Additionally, we suggest conducting future studies to estimate the predictive validity of the CBI-Br, in order to provide additional evidence related to the instrument validity.

## Data Availability

The datasets generated and analyzed during the current study are not publicly available due to the requirements of the Human Research Ethics Committee but are available from the corresponding author on reasonable request.

## Appendix

The Copenhagen Burnout Inventory–Brazilian version (CBI-Br)

**Table:**

**Burnout pessoal**	
*Personal burnout*	
1. Com que frequência se sente cansado?	
*How often do you feel tired?*	
2. Com que frequência se sente fisicamente exausto?	
*How often are you physically exhausted?*	
3. Com que frequência se sente emocionalmente exausto?	
*How often are you emotionally exhausted?*	
4. Com que frequência pensa “Não aguento mais”?	
*How often do you think: “I can’t take it anymore”?*	
5. Com que frequência se sente esgotado?	
*How often do you feel worn out?*	
6. Com que frequência se sente fraco e suscetível de adoecer?	
*How often do you feel weak and susceptible to illness?*	
**Burnout relacionado ao trabalho**	
*Work-related Burnout*	
7. Sente-se esgotado no final de um dia de trabalho?	
*Do you feel worn out at the end of the working day?*	
8. Sente-se exausto logo pela manhã quando pensa em mais um dia de trabalho? *Are you exhausted in the morning at the thought of another day at work?*	
9. Sente que cada hora de trabalho é cansativa para você?	
*Do you feel that every working hour is tiring for you?*	
10. Tem tempo e energia para a família e os amigos durante os momentos de lazer?	
*Do you have enough energy for family and friends during leisure time?*	
11. O seu trabalho é emocionalmente exaustivo?	
*Is your work emotionally exhausting?*	
12. Sente-se frustrado com o seu trabalho?	
*Does your work frustrate you?*	
13. Sente-se exausto de forma prolongada com o seu trabalho?	
*Do you feel burnt out because of your work?*	
**Burnout relacionado aos colegas**	
*Colleagues-related Burnout*	
14. Você acha difícil trabalhar com seus colegas?	
*Do you find it hard to work with colleagues?*	
15. Sente que esgota sua energia quando trabalha com colegas?	
*Does it drain your energy to work with colleagues?*	
16. Acha frustrante trabalhar com colegas?	
*Do you find it frustrating to work with colleagues?*	
17. Sente que dá mais do que recebe quando trabalha com colegas?	
*Do you feel that you give more than you get back when you work with colleagues?*	
18. Está cansado de aturar os colegas?	
*Are you tired of working with colleagues?*	
19. Alguma vez se questiona quanto tempo mais conseguirá trabalhar com os colegas?	
*Do you sometimes wonder how long you will be able to continue working with colleagues?*	

## Abbreviations

AVE: Average variance extracted
CB: Colleagues-related burnout
CBI: Copenhagen Burnout Inventory
CBI-Br: Copenhagen Burnout Inventory–Brazilian version
CBI-SS: Copenhagen Burnout Inventory–Students Survey
CFA: Confirmatory factor analysis
CFI: Comparative fit index
CR: Composite reliability
CVI: Content validity index
LM: Lagrange multipliers
ML: Maximum likelihood
PB: Personal burnout
RMSEA: Root mean square error of approximation
SOHM: Second-order hierarchical model
TLI: Tucker-Lewis index
WB: Work-related burnout
